# The influence of moisture ingression on mechanical and vibrational properties of basalt, glass, and hybrid fiber-epoxy composites

**DOI:** 10.1371/journal.pone.0360755

**Published:** 2026-10-21

**Authors:** Dayananda Pai, Jibin Jacob John, Sriharsha Hegde, I. V. Thara Reshma, N. H. Padmaraj

**Affiliations:** Manipal Institute of Technology, Manipal Academy of Higher Education, Manipal, India; King Mongkut’s University of Technology North Bangkok, THAILAND

## Abstract

Basalt fiber, a natural and eco-friendly reinforcement, has demonstrated superior properties compared with conventional synthetic fibers. This study investigates the effect of ageing on the mechanical and vibrational properties of Basalt/Epoxy, Glass/Epoxy, and Basalt-Glass/Epoxy hybrid composites. Laminates were fabricated using vacuum bagging, ensuring uniform fiber-matrix volume fractions, and then exposed to ageing in seawater, tap water, and distilled water for a duration of 80 days. The moisture absorption pattern followed Fickian diffusion, with seawater exhibiting the highest diffusion coefficients owing to matrix breakdown and osmotic influences. Tensile tests demonstrated a decrease in failure stress and modulus for all materials after ageing, with Glass/Epoxy composites experiencing the most notable degradation despite absorbing less moisture, indicating a weakening of the fiber-matrix interfacial bond. The hybrid laminates exhibited moderate tensile and vibrational characteristics and maintained mechanical performance better in harsh environments. Free vibration tests showed that the natural frequency diminished as mass increased due to moisture absorption, while damping factors rose after ageing. Overall, the combination of basalt and glass fibers enhanced environmental resilience, indicating their suitability for structural applications under challenging conditions.

## 1 Introduction

Composite materials are a combination of two or more constituent elements with different physical and chemical properties, such that the combined characteristics of the composite are better than the features of the individual constituents. In polymer-based composites, the combination of more than one fiber type is desired in a single composite, and hybrid composites are used, which are obtained by using two or more different types of fibers in a single matrix [1,2]. Commonly used reinforcements for polymer composites include fiberglass, carbon fiber, aramid fiber, and different types of natural fibers [3,4] For the past two decades, basalt fibers have been considered as a potential reinforcement for composites. Basalt fiber has excellent wear-resistant properties and can be used in several engineering and structural applications [5]. Basalt is a volcanic rock and is an ideal raw material as a reinforcement in polymer composites. The mechanical and chemical properties of these fibers are similar to those of glass fibers; however, they can be used over a wide temperature range without significant loss of mechanical properties [5,6]. Basalt fibers are biologically inert and environmentally friendly and can be used under extreme operating conditions. Owing to these properties, basalt fiber can be an alternative to asbestos and glass fibers. Basalt fibers contain mineral boundaries, pores, and discontinuities that act as stress-concentration sites, resulting in relatively lower tensile performance but better compressive properties [7].

Lopresto et al. [8] evaluated the quasi-static mechanical behaviour of basalt fiber–reinforced polymer (BFRP) and glass fiber–reinforced polymer (GFRP) composites. Their findings indicate that BFRP possesses a Young’s modulus approximately 35–42% higher than that of GFRP. Furthermore, BFRP demonstrated superior performance in both compressive and flexural strength. Dorigato and Pgoretti [9] compared the tensile and fatigue properties of basalt fiber-reinforced composites with glass fiber and carbon fiber composites. For specimens prepared by maintaining the same aerial density in all three composites, the basalt fiber showed better elastic moduli and strength compared to glass fiber composite, and its maximum tensile strength is at par with carbon fiber composite. The BFRP laminate exhibited better properties for sustaining progressive damage during fatigue testing.

Chen et al. [10] examined the quasi-static response of BFRP/epoxy composites and reported that their dynamic strength is roughly twice the corresponding quasi-static value. España et al. [11] investigated the effects of various coupling agents on basalt-fiber composites with bio-based resin matrices. They reported that silane-treated carbon/basalt hybrid composites exhibited superior tensile and flexural properties compared with composites containing untreated or acid-treated fibers. Liu et al. [12] evaluated the durability of BFRP composites subjected to saltwater immersion, moisture absorption, temperature fluctuations, and cyclic humidity. Their findings indicated that vinyl ester-based composites exhibited performance comparable to that of epoxy-based systems in a marine environment.

Song et al. [13] explored basalt fiber–reinforced PVDF/PMMA (70:30) composites and observed that both tensile and flexural strengths increased from 37 MPa to 62 MPa and from 41 MPa to 102 MPa, respectively, as fiber content rose to 20%, after which a reduction in properties occurred. Yao et al. [14] compared the dynamic tensile behaviour of unidirectional basalt, carbon, glass, and plain-woven aramid fibers, showing that carbon and aramid systems exhibit higher strength, stiffness, and toughness, while glass fibers demonstrate greater ductility. The values of basalt fabrics were at intermediate levels.

Vatsalya et al. [15] investigated the effects of seawater exposure on the mechanical performance of basalt-fiber-reinforced polymer (BFRP) composites. The basalt fibers were modified using 5% sodium bicarbonate and peroxide solutions to enhance their mechanical interlocking with the epoxy matrix. Chemical treatment increased resistance to moisture ingress by 28%. Furthermore, elastic modulus, tensile strength, and hardness improved by 45%, 31%, and 28%, respectively. Louis et al. [16] investigated the effect of seawater ageing for a duration of 7.5 years at 15°C and 40°C on Basalt/Epoxy composites. The ageing of basalt fibers in natural seawater resulted in the formation of iron oxide layers on the fiber surface. The mechanical properties of the BFRP composites rapidly degraded (40% at 15°C and 60% at 40°C) in the initial period of ageing. During the long-term ageing process, the overall degradation of the mechanical property was found to be 20% and Epoxy matrix acted as a protective layer for the core fibers. Fiore et al. [17] evaluated the behaviour of glass/epoxy laminates subjected to thermal cyclic ageing. The laminates were exposed to wet and dry environments for 30 and 21 days, respectively. The flexural strength and modulus of the laminates decreased by 20% and 10%, respectively. Mehdi et al. [18] conducted experiments by varying the stacking sequence and thermal cycling ageing to analyse the tensile behaviour of fiber metal laminates (FML) consisting of glass and basalt as reinforcements. The FML reinforced with four layers of basalt fiber exhibited better mechanical properties than the FML reinforced with glass and basalt fibers. The thermal stability of the FML consisting of Basalt fibers was better than that of the FMLs consisting of glass or hybrid reinforcements.

The available literature indicates that basalt fibers, derived from naturally occurring basalt rock, offer mechanical properties comparable to those of conventional synthetic fibers. Hybridising basalt fibers with other reinforcements, such as glass fibers, may improve the mechanical performance and environmental durability of polymer composites. Although several studies have examined the degradation of synthetic-fiber-reinforced composites, most have focused on single-fiber systems. Consequently, limited information is available on the combined effects of environmental ageing on the tensile and vibration characteristics of basalt/glass hybrid composites.

In particular, the relationships among moisture absorption, fiber–matrix interfacial degradation, tensile-property retention, natural frequency, and damping behaviour have not been systematically evaluated under identical manufacturing and ageing conditions. To address this research gap, the present study investigates basalt/epoxy, glass/epoxy, and basalt–glass/epoxy laminates exposed to different ageing environments. Moisture absorption, tensile properties, natural frequencies, and damping characteristics are evaluated using relevant ASTM standards.

The novelty of this work lies in the comparative assessment of single-fiber and hybrid laminates manufactured and aged under consistent conditions. By correlating moisture uptake with changes in tensile and vibration responses, the study provides an integrated understanding of how basalt–glass hybridisation influences the environmental durability and dynamic performance of epoxy composites.

## 2 Materials and Methods

The durability of fiber reinforced composites in harsh environments remains one of the biggest challenges in the marine and aerospace industry. The Basalt fiber is known for its high corrosion resistance and compatibility with polymer matrices. The present study focused on estimating the durability of the Basalt/Epoxy composites in different environments.

### 2.1 Specimen preparation

Bidirectional glass fiber with an aerial density of 360 GSM, procured from Hindustan Composite Solutions, India and bidirectional basalt fiber with an aerial density of 320 GSM obtained from Arrow Technical Textiles Pvt. Ltd., India was used as reinforcement. Medium viscosity epoxy resin Lapox L-12 (ARL-12) with low viscosity hardener Lapox K-6 (AH-312) supplied by Yuje Enterprises, Bangalore, was used as the matrix material. The material properties of the procured reinforcements are listed in Table 1.

**Table 1:** Properties of the Reinforcement Materials.

Property	Glass fiber	Basalt fiber
Density (*g*/*cm*^3^)	2.54	2.8
Area weight (*gsm*)	360	320
Thickness (*mm*)	0.4	0.32
Woven pattern	Bidirectional plain	Bidirectional plain

In the present study, three variants of laminates were fabricated using the vacuum bagging technique by reinforcing epoxy with basalt and glass fibers. Glass/Epoxy and Basalt/Epoxy laminates were fabricated by reinforcing 6 layers of respective fibers with epoxy (Fig 1). The hybrid laminates were fabricated by reinforcing four layers of Basalt and two layers of glass fabric in a stacking sequence of B/G/B/B/G/B as shown in Fig 1. In the hybrid composite, Basalt fabric was used as the outer layer due to better chemical and environmental resistance as compared to Glass fabric [19,20]. The method of manufacturing, specimen ageing, and test methods adopted in the study is shown in Fig 2. The weight fractions of the fiber and matrix in all the laminates were maintained at 40% and 60%, respectively.

**Fig 1:**
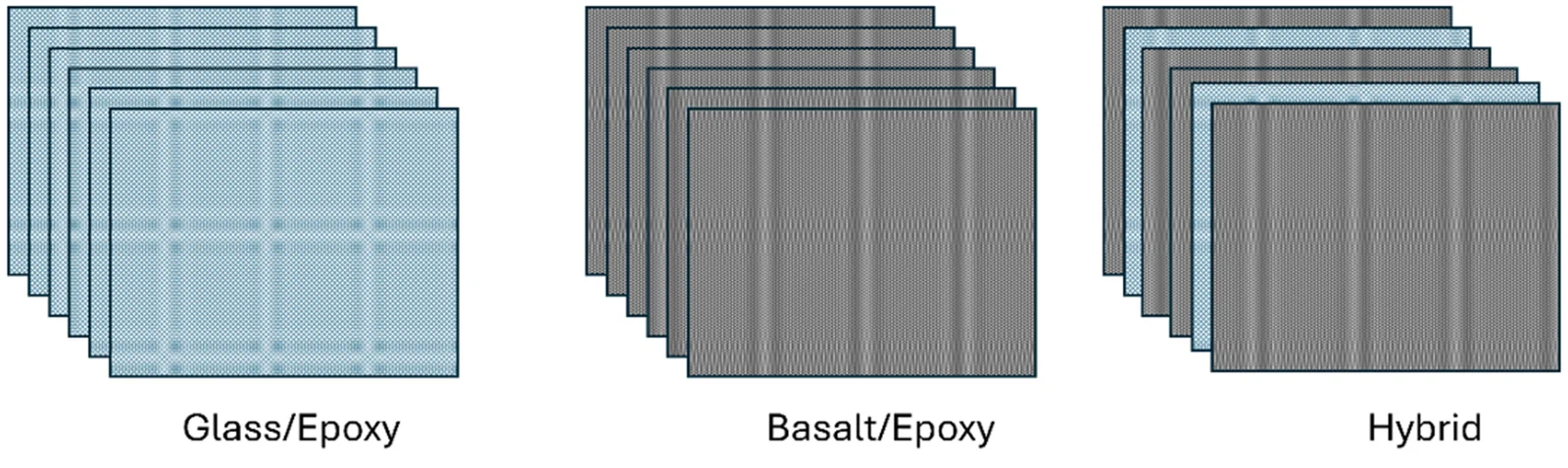
Stacking Sequence of the laminates.

**Fig 2:**
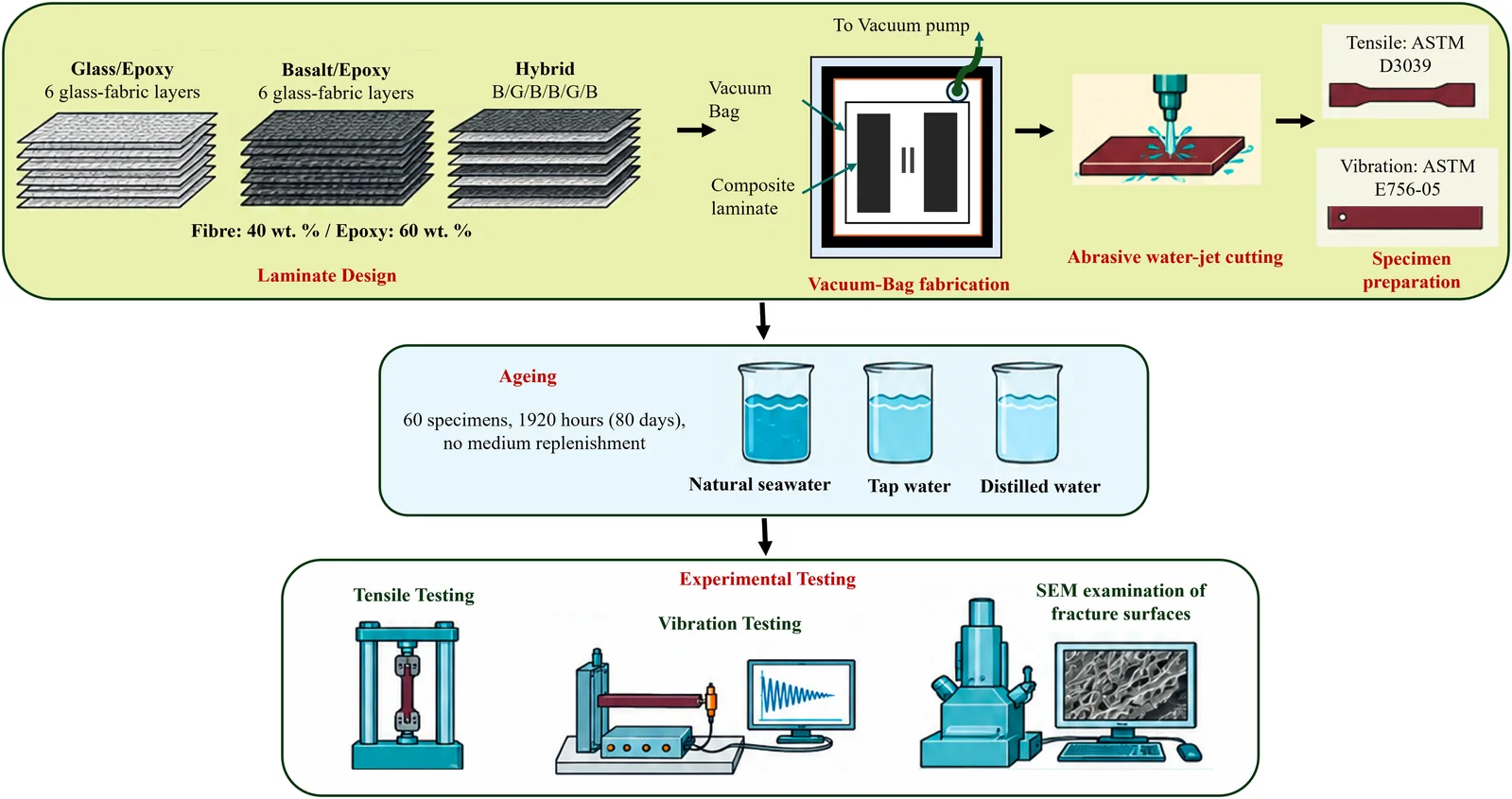
Specimen preparation, Ageing and Testing.

The laminate thickness of Glass/Epoxy, Basalt/Epoxy and Hybrid laminate were measured using a digital vernier caliper and the average thickness of the laminate was found to be 2.21 mm, 1.96 mm and 2.12 mm, respectively. The experimental and theoretical densities of the Glass/Epoxy, Basalt/Epoxy, and Hybrid laminate are measured using Archimedes’ principle and the Rule of Mixtures. Based on the theoretical and experimental densities, the void content of the laminates was computed. The measured theoretical density, experimental density and void content of the laminates are listed in Table 2.

**Table 2:** Density and Void estimation of the prepared laminates.

Specimen Type	Experimental Density (*g*/*cm*^3^)	Theoretical Density (*g*/*cm*^3^)	Void Content (%)
Basalt/Epoxy	1.52±0.018	1.57±0.009	3.18±0.01
Glass/Epoxy	1.48±0.005	1.52±0.007	2.23±0.07
Hybrid	1.49±0.011	1.53±0.014	2.61±0.04

The test specimens from the fabricated laminates were prepared as per ASTM 3039 and ASTM E 756−05 for tensile and vibration tests, respectively. The specimens of dimension 250 mm × 25 mm were cut from the laminate using abrasive water jet machining at Stonemax Machining Centre, Kochi, India.

### 2.2 Ageing process

The present study adopted three different ageing conditions namely, natural seawater, tap water and distilled water ageing. A sufficient volume of the ageing medium was taken during the study (Fig 2), without replenishing. Sixty specimens were subjected to ageing, out of which the weights of twelve were regularly monitored for 1920 hours (80 days). The current study mainly focused on understanding the effect of short-term ageing on hybrid composites and duration was selected based on the published literature [15,21,22]. A digital-weighing precision balance (Sartorius BSA2235) with an accuracy of 0.001 gram was used to weigh the specimens. The moisture absorption percentage was determined using the gravimetric method. Later, the diffusion coefficient for each condition was calculated using Eq (1) [23,24].


$$\begin{array}{c} D=\pi (\frac{h}{4M_{\infty }}{)}^{2}(\frac{\left(M_{2}-M_{1}\right)}{\sqrt{T_{2}}-\sqrt{T_{1}}}{)}^{2} \end{array}$$ (1)

Where,

*D*: Diffusion Coefficient (*m*^2^/*s*)

*h*: Thickness of the specimen (*m*)

$M_{\infty }$: Moisture absorption percentage at saturation state

$M_{i}$: Moisture content at time $T_{i}$

*T*_1_ and *T*_2_: Time intervals (*sec*)

### 2.3 Mechanical and vibration test

As part of the mechanical analysis, the pristine and aged specimens were subjected to a quasi-static tension test. The tensile tests were conducted based on ASTM D3039 [25] using a BiSS-make 50 kN capacity Universal Testing Machine (UTM). The test specimens were gripped using a mechanical wedge grip, with 30 mm of the specimen as grip length, and the load was applied at a rate of 0.016 kN/s. The average failure stress and average Young’s modulus were determined from multiple experimental data obtained from the tensile tests. In each condition, five specimens were used for the test to evaluate the average tensile properties.

The dynamic characteristics of the pristine and aged composites were studied by identifying their natural frequencies and stiffness using free-vibration testing. An accelerometer of sensitivity 100.6 mV/g and four-channel NI 9234 Data Acquisition System (DAQ) procured from National Instruments were used for this purpose. Three trials of free vibration test were conducted in each category of specimen by mounting the specimen in a fixed free condition (cantilever). Time domain data were extracted from the free-vibration setup and used to calculate the damping ratio from logarithmic decay (time domain curve). During the test, excitation was applied by tapping the free end of the specimen. The stiffness of the pristine and aged specimens was estimated based on Eq (2) [26].


$$\begin{array}{c} w_{n}=\sqrt{\frac{k}{m}} \end{array}$$ (2)

Where

$w_{n}$: Natural frequency (*rad*/*s*)

*k*: Stiffness (*N*/*m*)

*m*: Mass of the specimen (*kg*)

## 3 Results

### 3.1 Moisture absorption behaviour, and diffusion coefficient

The moisture absorption behaviour of each type of specimens during the ageing process was observed up to 1920 hours (80 days). The moisture absorption curve of the Basalt/Epoxy, Glass/Epoxy and Hybrid specimens are shown in Fig 3.

**Fig 3:**
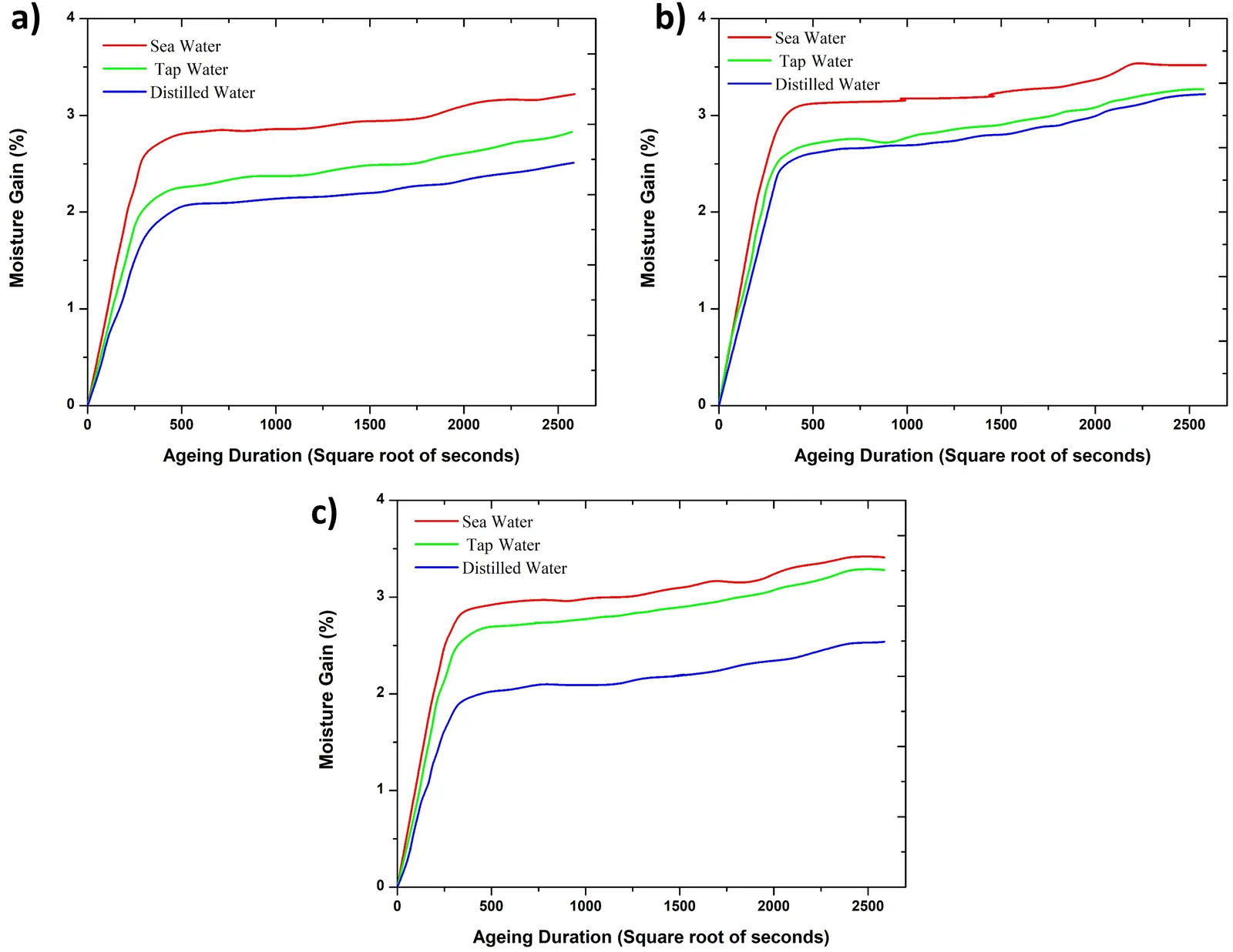
Moisture Absorption Behaviour: a) Basalt/Epoxy; b) Glass/Epoxy; c) Hybrid Specimens.

The moisture absorption curve of Basalt/Epoxy, Glass/Epoxy and Hybrid specimens followed a Fickian diffusion curve under all ageing conditions. In the initial ageing period (approximately 70 hours) all three types of specimens exhibited rapid moisture intake. In the second stage, the moisture absorption rate became steady, and specimens eventually reached an equilibrium state by the end of the ageing process.

The moisture intake in all three cases was influenced by the ageing medium. The salt content of the seawater reacts with the matrix material and tends to reduce the stability of the epoxy. Additionally, the higher ionic concentration of seawater induces osmotic gradient during the ageing process. The combined effect of sea salt and osmotic pressure can lead to formation and propagation of micro cracks on the specimen surface [27,28]. These reactions can further weaken the cross-linking within the matrix. This combined effect increases the diffusion coefficient (Fig 4) of specimens aged in seawater, leading to increased moisture absorption.

**Fig 4:**
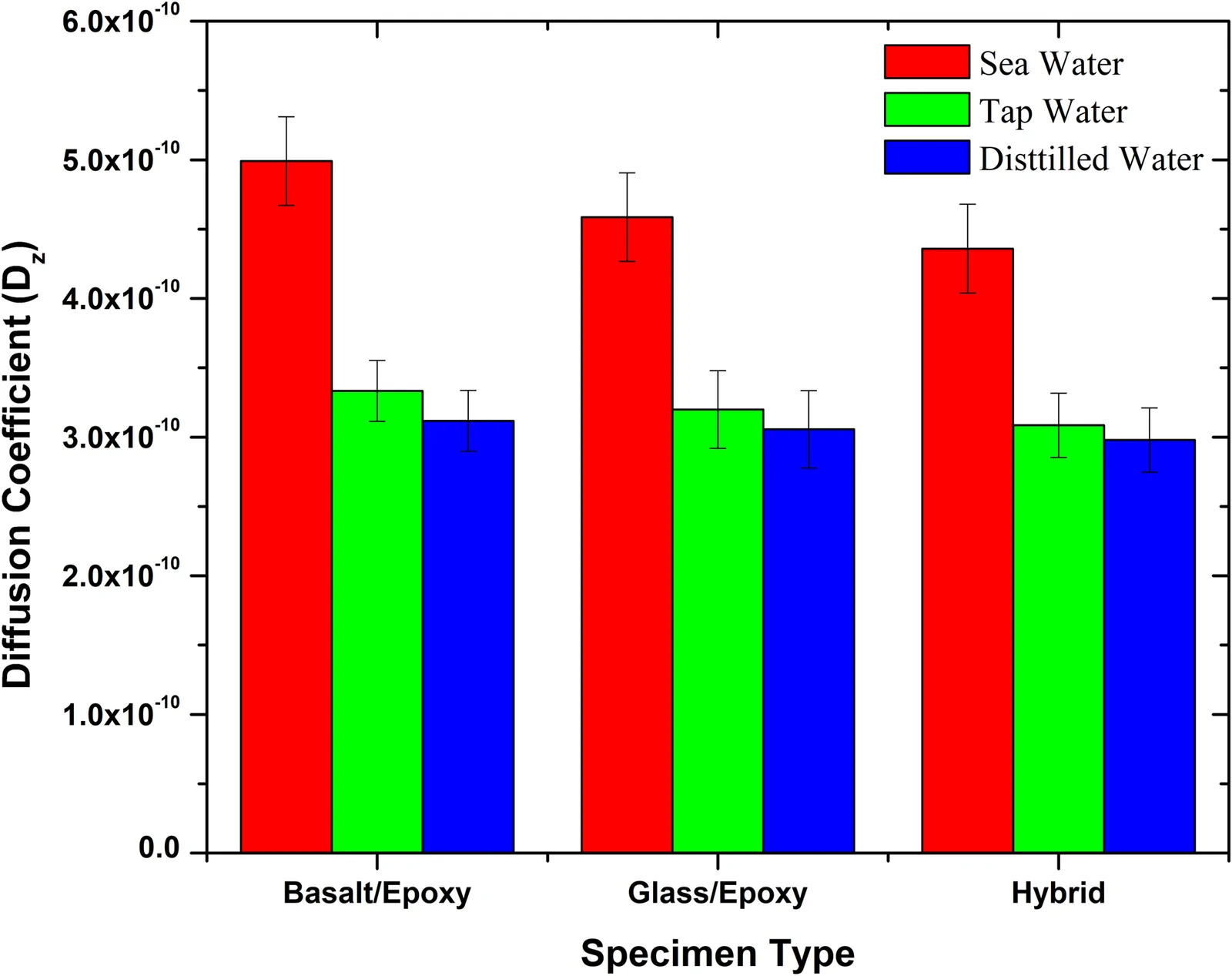
Diffusion Coefficient of the specimens in different ageing conditions.

In contrast, tap water and distilled water contain fewer salts, which reduces the chemical interaction between the matrix and the ageing medium. As a result, lower diffusion coefficients were observed (Fig 4) compared to that of the seawater ageing process. The analysis of diffusion coefficients and the moisture gain curve clearly indicates that the moisture intake behaviour of the specimens mainly depended on the matrix properties rather than the reinforcement. The results also show that the moisture intake is influenced by the void content of the specimens. The void content of the Basalt/Epoxy was found to be higher than the other two categories (Table 2), resulting in higher moisture intake in Basalt/Epoxy laminates.

### 3.2 Effect of ageing media on tensile properties

Quasi-static tensile test was conducted on pristine and aged Basalt/Epoxy, Glass/Epoxy and Hybrid as per ASTM 3039 standard. Five specimens from each category were tested, and the average values of tensile strength and modulus were estimated. The stress-strain plots of the representative specimens from each category are presented in Fig 5 (a), (b), and (c), respectively. The stress-strain curve of all the specimens exhibited a bilinear nature, characterized by two distinct slopes. This behaviour represents the different failure points of the 90° and 0° oriented fibers in the individual lamina. The transition point in the deformation behaviour, known as the knee point, was observed in all three types of specimens [29]. The variation of tensile properties of all the three specimens are shown in Fig 5 (a), (b), and (c), respectively. In all the categories, pristine specimens showed the highest average failure stress as compared to their aged counterparts. The pristine Basalt/Epoxy specimens failed at an average stress of 228.52±12.36 MPa.

**Fig 5:**
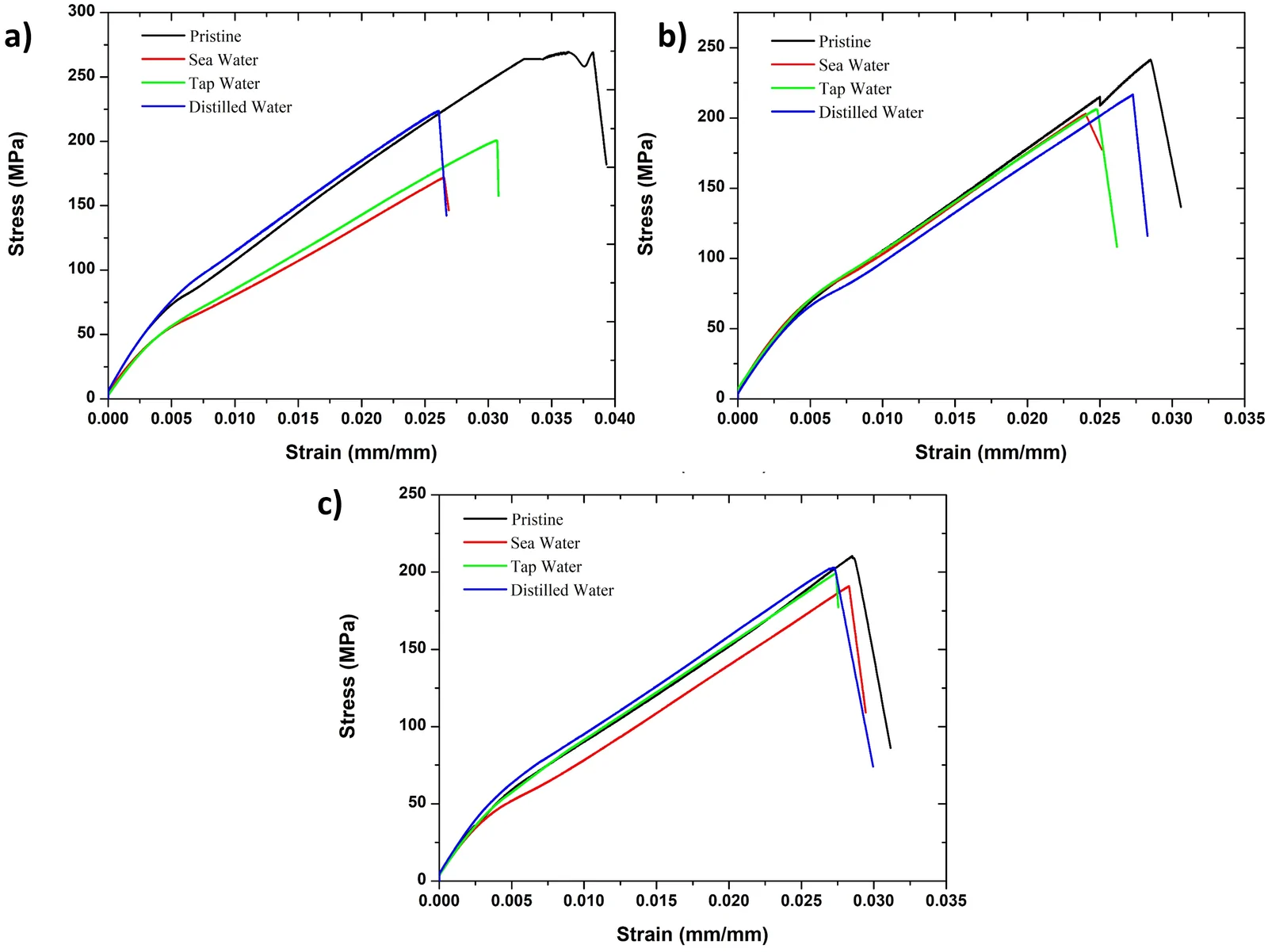
Stress-Strain curve: a) Basalt/Epoxy; b) Glass/Epoxy; c) Hybrid Specimens.

Ageing of Basalt/Epoxy laminates led to a reduction in failure stress due to an increase in moisture absorption. The specimens aged in seawater, tap water and distilled water failed at average failure stresses of 192.42±8.48, 196.83±12.45 and 202.26±13.87 MPa, respectively. The higher moisture gain in the specimens resulting in the weakening of the matrix/fiber interphase and thereby reducing the load-carrying capacity compared to the pristine specimens. As compared to the pristine specimens, ageing in seawater, tap water and distilled water reduced the average failure stress by 15.84%, 14.23% and 10.08%, respectively. The stress-strain behaviour of the pristine and aged Glass/Epoxy specimens is shown in Fig 5 (b). The tensile test clearly showed that the mechanical properties of the Glass/Epoxy specimens were superior to the Basalt/Epoxy specimens, with a 10.66% increment in the tensile strength. However, ageing had a more significant effect on Glass/Epoxy laminates; ageing in seawater reduced the tensile strength by 36.17% in comparison to pristine specimens, while ageing in tap water and distilled water reduced the tensile strength by 25.44% and 16.97%, respectively, with respect to the pristine specimens. Although the moisture ingression or the diffusion coefficient in the Glass/Epoxy was lower, it drastically reduced the mechanical property. The moisture ingress reduced the interfacial bonding between the Glass and Epoxy resulting in poor stress transfer from the matrix to the fiber surface.

The stress-strain behaviour of hybrid pristine and aged specimens is represented in Fig 5(c). The hybrid pristine and aged specimens exhibited lower tensile properties as compared to Basalt/Epoxy and Glass/Epoxy specimens. The pristine hybrid specimens failed at an average stress of 209.54±8.38 MPa, which was lower than that of Basalt/Epoxy and Glass/Epoxy specimens by 12.88% and 22.31%, respectively. Ageing of hybrid composites in seawater, tap water and distilled water reduced the tensile strength by 9.21%, 5.23% and 3.43%, respectively.

The variation of Young’s Modulus with respect to different ageing conditions for all three categories of specimens is shown in Fig 6. The modulus of each specimen was estimated below the knee point to get the overall modulus of the material. The modulus depended on the type of reinforcement, with Glass/Epoxy specimens exhibiting higher values compared to Basalt/Epoxy and Hybrid composites. The analysis also showed that the modulus of the Glass/Epoxy specimens depended on the ageing conditions. The modulus of the pristine specimen was 15.36±0.85 GPa, and for seawater, tap water and distilled water aged specimens, it was 11.52±0.47, 12.52±0.78 and 13.34±0.97 GPa, respectively. Similarly, the modulus of the pristine Basalt/Epoxy specimens was found to be 11.24±0.34 GPa, and for seawater, tap water and distilled water aged specimens, it was estimated as 9.34±0.28, 10.08±0.56 and 10.97±0.74 GPa, respectively.

**Fig 6:**
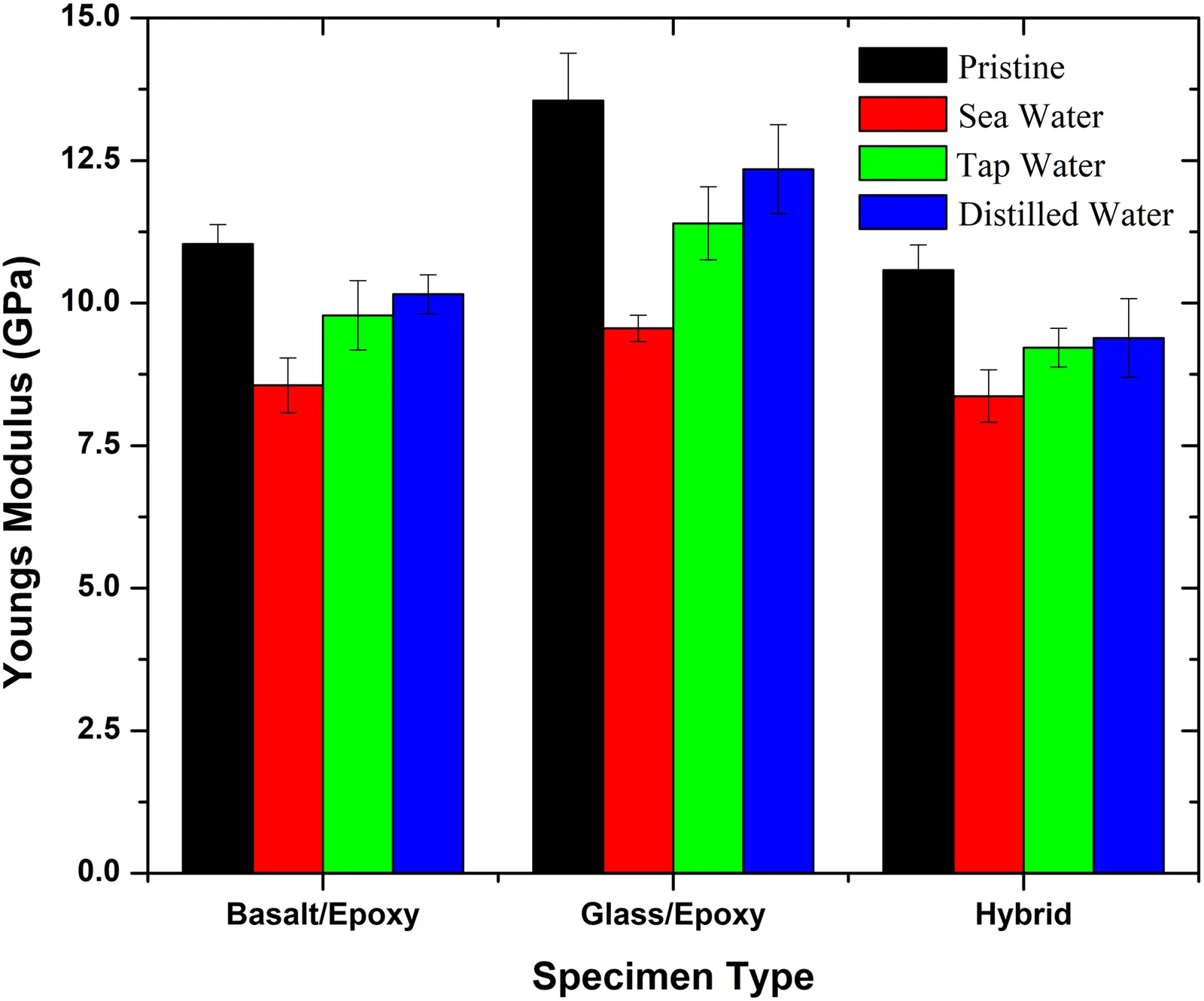
Effect of ageing condition on Youngs’ Modulus of the specimens.

The hybrid specimens showed intermediate properties compared to the two other types. The modulus of the pristine specimen was calculated to be 14.8±0.95 GPa. The moisture intake in hybrid specimens was comparatively low, and this aided in retaining the modulus of the material in different ageing conditions. Seawater-aged hybrid specimens showed a modulus of 12.81±0.68 GPa, retaining 86.5% of modulus compared to pristine specimens. The modulus of the tap water and distilled water aged specimens were 13.45±0.36 and 14.01±0.79 GPa, respectively. Compared to the pristine hybrid specimens, ageing in tap water and distilled water reduced the modulus by 9.21% and 5.33%, respectively.

During seawater ageing, dissolved salts penetrated the microcavities and voids within the specimens. Their subsequent crystallisation generated localised stresses, thereby promoting damage initiation and propagation. In contrast, exposure to tap and distilled water primarily caused hydrolysis and matrix plasticisation. Specimens aged in these media exhibited better retention of mechanical properties than those exposed to seawater, owing to the absence of salt deposition and crystallisation. These findings may support the design of lightweight composite components for applications exposed to severe environmental conditions, including boat hulls, offshore wind-turbine blades, surfboards, and other marine or outdoor structures [15] [30].

### 3.3 Effect of ageing media on natural frequency

The effect of ageing on the natural frequency of Glass/Epoxy, Basalt/Epoxy and Hybrid specimens was studied using free vibration tests (Eq 2). Fig 7 shows the variation in natural frequency of different types of specimens under various ageing media. The natural frequency of the material depends on its stiffness and density. The natural frequency of the pristine Glass/Epoxy and Basalt/Epoxy were found to be 11.24 Hz and 15.36 Hz, respectively. This difference is mainly due to the lower density of the Glass/Epoxy specimens (Table 2). Hybridization of Basalt with Glass resulted in an increase in both the mass and density of the specimens as compared to the Glass/Epoxy, resulting in an intermediate natural frequency (14.8 Hz) for the hybrid composites. The mass and density of all specimens increased due to the ageing process in different conditions. As the mass gain increased due to moisture intake, the natural frequency of all the tested specimens decreased. The specimens aged in seawater absorbed the highest amount of moisture and exhibited the lowest natural frequency.

**Fig 7:**
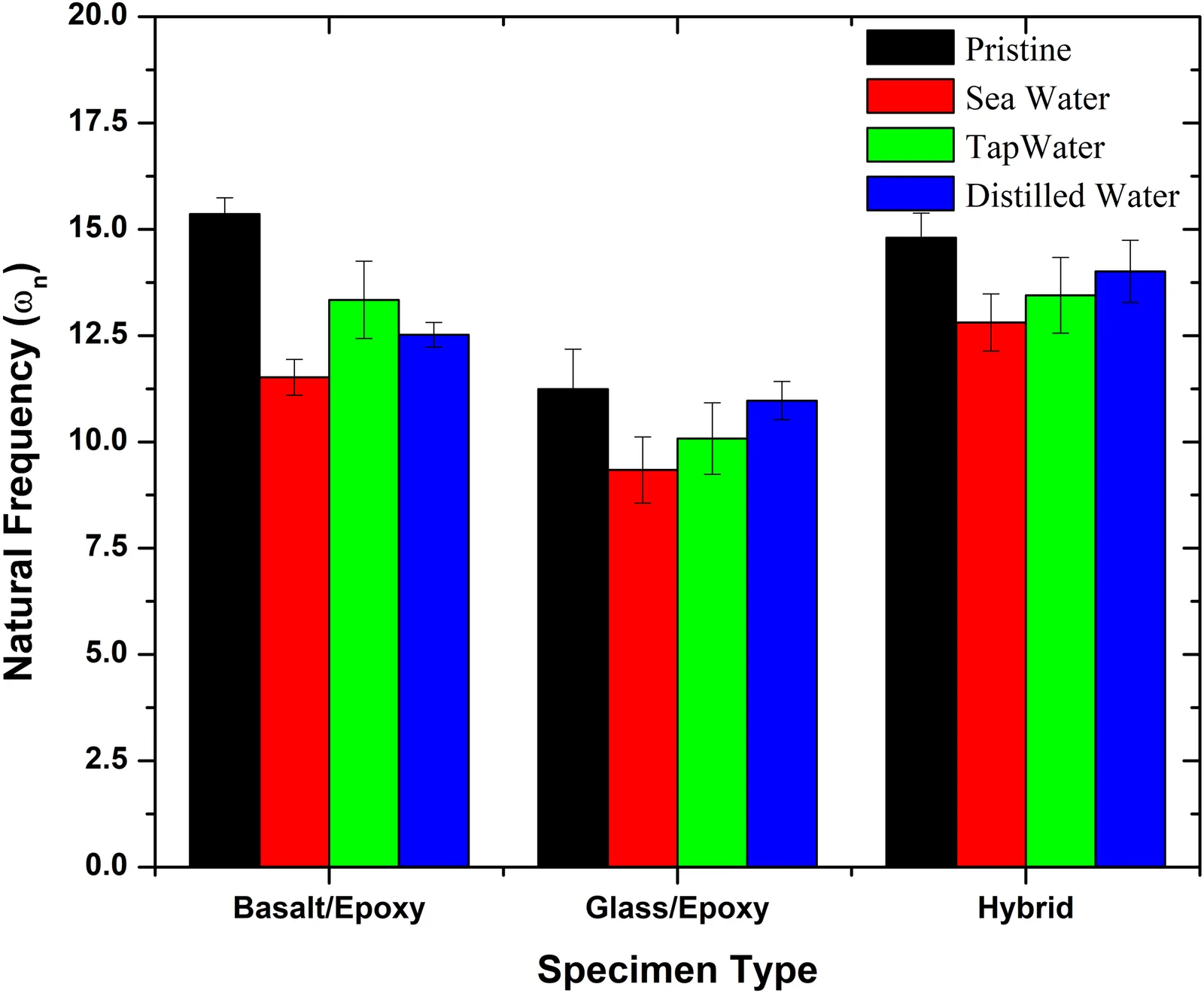
Effect of ageing condition on natural frequency of the specimens.

It has been observed that as ageing progresses, the specimens absorb moisture, resulting in a gradual reduction in stiffness. This trend is evident from Fig 6, which depicts the reduction of Young’s modulus with ageing. For vibration tests, a fixed-free boundary condition was employed, for which the stiffness is calculated using Eq. (3). Hence, it is clear that the natural frequency of the specimens decreased progressively with ageing under all environmental conditions. This reduction is attributed to the deterioration of the elastic modulus and the consequent loss of structural stiffness in different ageing conditions.


$$\begin{array}{c} k=\frac{3EI}{L^{3}} \end{array}$$ (3)

Where

*E*:Young’s Modulus (*GPa*)

*I*: Moment of Inertia (*kgm*^2^)

*L*: Length of the beam (*m*)

### 3.4 Effect of ageing media on damping factor

The damping factor of pristine and aged Glass/Epoxy, Basalt/Epoxy and Hybrid specimens was estimated from free-vibration test data using the logarithmic decrement method. The variation of damping factor with respect to types of reinforcement and ageing condition is shown in Fig 8.

**Fig 8:**
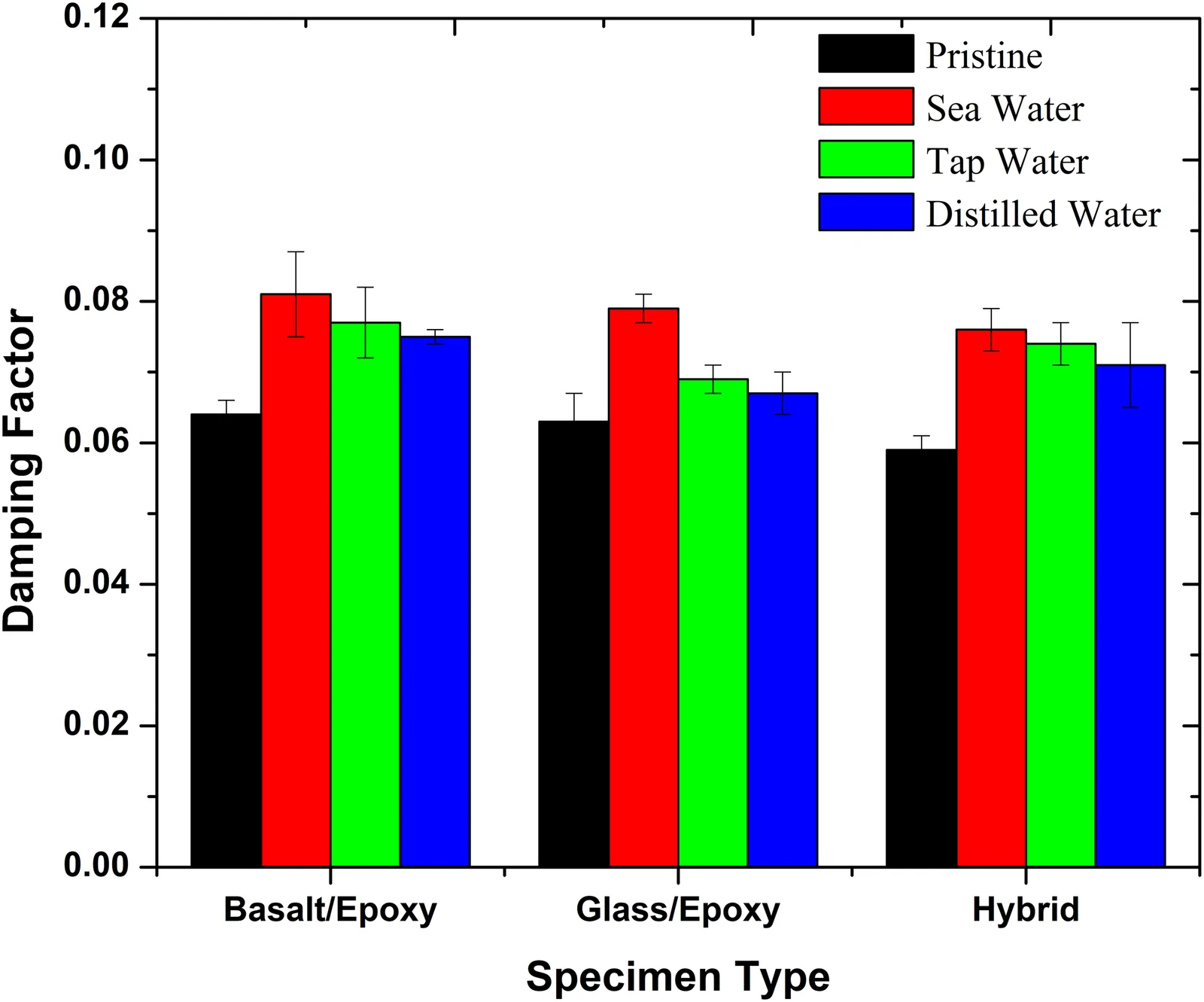
Effect of ageing condition on damping factor of the specimens.

The analysis of the data obtained from the free-vibration test showed that pristine Glass/Epoxy, Basalt/Epoxy, and Hybrid specimens showed a similar trend, with damping factors of all specimens being less than 0.1. This is mainly due to uniform bonding between the fiber and matrix materials [31,32]. The damping behaviour of the composite is due to the inherent damping properties of the matrix, the fiber reinforcement and the fiber-matrix interaction during the vibration.

Additionally, the void content of the Glass/Epoxy, Basalt/Epoxy and Hybrid specimens were found to be almost similar (Table 2). The presence of these voids induces friction between adjacent layers, thereby increasing the energy dissipation during the free-vibration test. Ageing of specimens under different conditions increased the damping factor as compared to the respective pristine specimens. Ageing of polymer-based composites resulted in the plasticization of the matrix material and degradation of the fiber-matrix interphase, resulting in discontinuities between fiber and matrix. This, in turn, increases the fiber-matrix interaction, thereby resulting in enhanced damping due to moisture ingression. As a consequence, the energy dissipation rate during the vibration test increases, leading to a higher damping factor. Among all ageing media, seawater-aged specimens showed the highest damping factors compared to others [31,33].

### 3.5 Failure surface morphology

The ageing of Glass/Epoxy, Basalt/Epoxy and hybrid specimens in seawater, tap water and distilled water varied both the mechanical and vibrational properties of the materials. Among these, the seawater-aged specimens had the most significant effect. The Scanning Electron Microscopy (SEM) images of seawater-aged tensile specimens were analysed to understand the structural changes at the fiber-matrix interphase. Fig 9 (a), (b) and (c) show the SEM images of the seawater aged Basalt/Epoxy, Glass/Epoxy and Hybrid tensile specimens, respectively. The application of tensile load on the seawater-aged Basalt/Epoxy specimens induced fiber-dominated failure characterized by complete fiber fracture (Fig 9(a)). A clean Basalt fiber breakage along the length of the fiber was observed. Additionally, debonding of matrix material from the fiber surface was observed, with multiple matrix flakes visible in the failure region. This effect is primarily caused by internal stresses that arise within the matrix as moisture penetrates the material during ageing [15,34].

**Fig 9:**
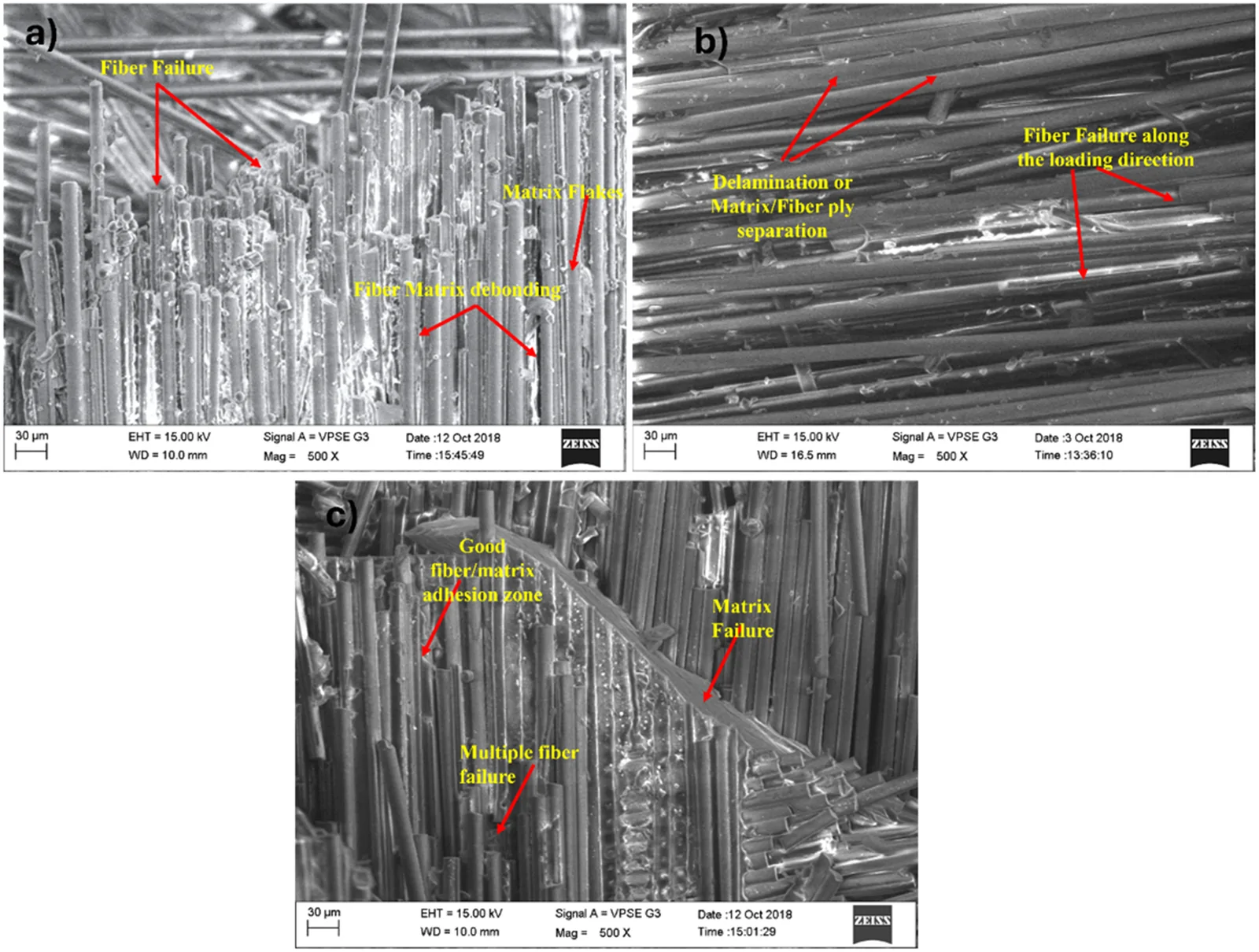
Failure morphology of seawater aged tensile specimens: a) Basalt/Epoxy; b) Glass/Epoxy and c) Hybrid Specimens.

The seawater ageing significantly reduced the tensile properties of Glass/Epoxy specimens (Fig 5(b)). The ingress of moisture and salt ions deteriorated the fiber-matrix interphase, resulting in a complete peeling of the matrix material from the failure region, as shown in Fig 9(b). Multiple Glass fiber fracture points were observed at different locations along the fiber length. The failure surface of Hybrid composites (Fig 9(c)) showed comparatively better fiber-matrix bonding. The failure was mainly composed of the fiber failure along the loading direction. Multiple shear bands and diagonal cracks were observed in the matrix failure region. Such matrix failure patterns can initiate delamination, resulting in multiple fiber failure points in alternate layers of the specimen.

## 4 Conclusions

The current research thoroughly investigated the mechanical and vibrational characteristics of Basalt/Epoxy, Glass/Epoxy, and Hybrid fiber-reinforced composites subjected to different ageing conditions, including exposure to seawater, tap water, and distilled water. The findings clearly indicated that, although all composites undergo property degradation due to moisture absorption and environmental exposure, their performance significantly differs depending on the type of fiber and the stacking sequence. Basalt/Epoxy composites, despite absorbing more moisture, retained 84.16% of their tensile strength and showed superior modulus retention during ageing, making them a promising option for challenging environments. Glass/Epoxy composites, although initially robust, were more prone to degradation, particularly in saline environments, with a 36.17% reduction in tensile properties as compared to pristine specimens. Hybrid composites exhibited a balanced behaviour, effectively maintaining both mechanical and vibrational properties, and showing good resilience to environmental degradation. Overall, the results support the use of hybrid reinforcement strategies to enhance the durability and performance of polymer matrix composites in applications prone to ageing, such as marine and structural components.

The main contribution of this study lies in comparing the moisture absorption, tensile response, and vibration behaviour of Basalt/Epoxy, Glass/Epoxy, and Basalt–Glass/Epoxy laminates prepared and aged under the same conditions. The findings show that combining basalt and glass fibres helps achieve a better balance between the retention of mechanical properties and vibration performance than using either fibre system alone. These results can assist in selecting and designing composite laminates for structural and marine components that are regularly exposed to moisture.

In the current study, ageing was conducted in three aqueous media for a maximum duration of 80 days. Future studies could examine long-term environmental degradation under different salinity, temperature, and pH conditions. The investigation was also restricted to selected fiber configurations, stacking sequences, and fiber-volume fractions; therefore, a broader range of laminate designs should be evaluated. Furthermore, machine-learning models could be developed using comprehensive experimental datasets to predict long-term ageing behaviour and the associated deterioration of material properties [35,36].

## Abbreviations

AH: aliphatic hardener
ARL: araldite
ASTM: American society for testing and materials
BFRP: basalt fiber–reinforced polymer
DAQ: data acquisition
FML: fiber metal laminates
GFRP: glass fiber–reinforced polymer
GSM: grams per square meter
NI: national instruments
PMMA: poly methyl methacrylate
PVDF: polyvinylidene fluoride
SEM: scanning electron microscope
UTM: universal testing machine
